# A 5‐year retrospective study of canine and feline patients referred to an isolation unit for infectious diseases

**DOI:** 10.1002/vro2.5

**Published:** 2021-04-05

**Authors:** Catarina Paulo, Inês Machado, Helena Carvalho, Joana Gomes, Ana Deodato Mota, Luís Tavares, Virgílio Almeida, Solange Gil

**Affiliations:** ^1^ Faculty of Veterinary Medicine University of Lisbon, Av. Universidade Técnica Lisbon Portugal; ^2^ CIISA‐ Centre for Interdisciplinary Research in Animal Health Faculty of Veterinary Medicine Av. Universidade Técnica University of Lisbon Lisbon Portugal; ^3^ Centro de Investigação e Estudos de Sociologia, University Institute of Lisbon (ISCTE ‐ IUL) Lisboa Portugal; ^4^ Veterinary Teaching Hospital Faculty of Veterinary Medicine University of Lisbon Lisbon Portugal; ^5^ Laranjeiras’ Veterinary Hospital Rua São Tomás de Aquino, 8C Lisbon 1600‐203 Portugal

**Keywords:** cat, dog, infectious diseases, isolation unit, patient referral

## Abstract

**Background:**

Referral of cases is becoming more and more frequent in companion animal practice. The Infectious Diseases Isolation Unit (IDIU) admits first opinion, second opinion and referred patients with a confirmed infectious disease (ID) or a clinically suspected ID that is awaiting laboratory diagnosis. The primary aims of this study were to describe the annual number and characteristics of patients referred to the IDIU and identify the most frequent IDs in referred dogs and cats. A secondary aim was to investigate possible differences in the length of the hospitalisation and the clinical outcome among referred cases and those admitted to the IDIU after first and second opinion appointments.

**Methods:**

A retrospective study was carried out on patients hospitalised at the unit over 5 years from 9th October 2013 to 31st December 2018.

**Results:**

The study population consisted of 365 dogs and 515 cats to give a total of 880 patients hospitalised at the IDIU from October 2013 to December 2018. Among the 96 referred dogs, parvovirosis (37.7%) and leptospirosis (31.1%) were the most frequent IDs. Feline upper respiratory tract infection (38.2%) and feline leukaemia virus infections (36.4%) were the main causes in the 80 referred cats. Worrying noncompliance rates of dog (51.0%) and cat (52.5%) vaccination schedules were identified. The analysis of the length of hospitalisation in the three groups of patients was not statistically different. In both animal species there were statistically significant higher clinical discharge rates on the first opinion patients’ group in comparison to referred patients and the second opinion group.

**Conclusions:**

Parvovirosis and leptospirosis in dogs and upper respiratory disease and feline leukaemia virus infection in cats were the most common diagnoses for patients admitted to the IDIU, reinforcing the need for accurate vaccination. Discharge rates results pinpoint the need for timely accurate reference.

**Abbreviations**
ABCD: European Advisory Board on Cat Diseases
FeLV: feline leukaemia virus
FIP: feline infectious peritonitis
FIV: feline immunodeficiency virus
HEPA: high efficiency particulate air
ID: infectious disease
IDIU: infectious disease isolation unit
IU: isolation units
MDR: multidrug‐resistant
PPE: personal protective equipment
SOP: standard operating procedures
URTI: (feline) upper respiratory tract infection
VTH: Veterinary Teaching Hospital
WSAVA: World Small Animal Veterinary Association

## BACKGROUND

A complex interplay between patients, animal health care workers and microorganisms occurs in the hospital environment, facilitating the transmission of infectious agents and creating a threat to both animal and public health.^1^ This chain of transmission may occur in three ways: animal‐to‐animal, animal‐to‐human and human‐to‐animal.^2^, ^3^, ^4^, ^5^ Veterinary professionals face an increase in the occurrence of hospital‐acquired infections which directly or indirectly have a negative financial, social and environmental impact on patients, staff and clients.^3^, ^6^, ^7^, ^8^ According to Stull et al,^6^ without the implementation of an effective infection control, prevention and biosecurity plan, it is not possible to ensure a safe and controlled hospital environment. Therefore, to protect animal health and welfare, as well as public health, all veterinary hospitals and clinics should run a specific and effective infection control plan.^3^, ^5^, ^6^, ^7^ Those plans are based on standard operating procedures (SOP), whose application provides a safe working environment and forms a hierarchy of effectiveness: hazard elimination (microbial contamination), design of hospital facilities, administrative controls and use of personal protective equipment (PPE).^6^

Isolation units were created with the main purpose of containing infectious agents in a specific area, avoiding their dissemination to other hospital areas and contamination of susceptible patients and staff.^1^, ^8^, ^9^ In order to achieve this goal, the early detection of high‐risk patients is crucial and, whenever possible, before arriving at the hospital.^1^, ^2^, ^10^ Thus, animals with confirmed or suspected infectious diseases (ID) should be directly transferred into the hospital's IU where they can be examined and treated under safe conditions and according to the SOP in force at the IU.^1^, ^2^, ^9^, ^10^ The Infectious Diseases Isolation Unit (IDIU or unit) of the Veterinary Teaching Hospital (VTH) of the Faculty of Veterinary Medicine of the University of Lisbon is a multispecies facility that admits animals with confirmed or suspected IDs. IDIU has a ward for dogs, another for cats, a ward for feline immunodeficiency virus (FIV) positive cats only and a ward for large animals, an antechamber, a workroom for animal health care workers and a storage room.^11^ All rooms work autonomously under negative air pressure, with a ventilation system, HEPA filters and a video surveillance system.^11^ Users of the unit put on PPE prior to handle patients and act according to a specific SOP.^11^ Referral of cases is becoming more and more frequent in companion animal practice.^12^ The veterinarian's decision to refer to a specialist is dependent upon several conditions including the need for expertise and guidance, additional equipment or services that are not available at the referring veterinary clinic, a definitive diagnosis and/or 24‐hour medical supervision; additional considerations may include worsening of the patient's medical condition and client disappointment with case progression.^13^ The age of the veterinarian seems to be a determining factor in the timeliness of referring a patient; younger veterinarians tend to refer their patients later, after they have carried out several diagnostic and/or treatment procedures or when the severity of the patient's condition reaches a critical point.^14^ Timing of referral is crucial as a delay in referring the patient may result in prolonged hospitalisation, worse prognosis or even death.^12^ When selecting an individual veterinary specialist or a referral veterinary hospital, the referring veterinarian should give priority to a specialist with whom he/she has good communication and that he/she fully trusts.^12^, ^15^ The referring veterinarian and the specialist should have an initial conversation to understand the referring veterinarian's expectations.^12^ These may be related to the skills and services the specialist can offer, the medical care to be provided along with the associated costs and to assign responsibilities to each veterinarian.^13^ The veterinary specialist needs access to the patient's history^13^, ^15^ and all diagnostic tests performed.^12^, ^13^, ^15^ After the first appointment, the specialist should inform the referring veterinarian about the definitive diagnosis and proposed treatment.^13^, ^15^ During patient hospitalisation, the specialist should keep the referring veterinarian updated on case progression.^12^, ^13^ The specialist should only provide the services requested by the referring veterinarian and should consult the referring veterinarian if other procedures become necessary.^13^, ^15^ In order to achieve the best patient care, it is extremely important that both veterinarians work as a team, sharing their knowledge and experience, to reach a joint decision with the owner regarding the best approach to take in each case.^12^ After patient discharge, the specialist should send to the referring veterinarian a detailed report advising on the patient follow‐up schedule.^13^, ^15^ The effectiveness of this professional interaction improves patient care, enhances client satisfaction and, consequently, increases the financial income of the medical institutions involved and fulfils their missions.^12^, ^13^

The primary aims of this study were to describe the annual number and characteristics of patients referred to the IDIU and identify the most frequent IDs in referred dogs and cats. A secondary aim was to investigate possible differences in the length of the hospitalisation and the clinical outcome among referred cases and those admitted to the unit after first and second opinion appointments.

## MATERIAL AND METHODS

A retrospective study was carried out on patients hospitalized at the IDIU over 5 years from 9th October 2013 to 31st December 2018. The study population consisted of dogs and cats that were admitted to the IDIU following either a first opinion appointment or second opinion appointment at the VTH or referral by a veterinarian of other veterinary clinics, hospital, public or private animal shelter. Data were collected from the management software of the VTH (Guruvet), IDIU database (in MS Excel 2016) and patients’ paper medical records. All animals that participated in this study were client‐owned and joined the study after owner's written consent and Ethical Committee approval. Collected data included patient‐related parameters such as animal species, sex, neuter status, age and vaccination status. Vaccination status was established based upon the Vaccination Guidelines Group of the World Small Animal Veterinary Association^16^ and the matrix vaccination guidelines of the European Advisory Board on Cat Diseases,^17^ updated in 2017.^18^ Vaccination status was coded using five categories:

- Complete vaccination status: dogs and cats that received the initial core vaccination at 6–8 weeks of age, then every 2–4 weeks until 16 weeks of age or older (primovaccination). A booster dose of vaccine given at 12‐month of age and thereafter, revaccinations every 3 years. The core vaccines for dogs are those that protect against canine parvovirus type 2 (CPV‐2), canine distemper virus (CDV) and canine adenovirus type 1 (CAV‐1). Regarding cats, the core vaccines are those that protect against feline panleukopenia virus (FPV), feline herpesvirus type 1 (FHV‐1) and feline calicivirus (FCV).
- Incomplete primovaccination status: all dogs and cats that were not old enough to have completed the primovaccination or any dog or cat missing one or more doses of the primovaccination.
- Delayed or interrupted scheduling of vaccination: Any dog or cat missing the 12‐month booster or any core revaccination every 3 years.
- Never vaccinated.
- Unknown vaccination status: animal with unknown vaccination history.

Other parameters included information regarding patient hospitalisation and included the annual number of referred patients, clinical presentation, presence of concomitant non‐IDs and definitive diagnosis of the ID.

### Statistical analysis

Descriptive statistics are reported as number (N) and per cent (%) and the median to characterize the central tendency of some variables. A comparative analysis was performed using nonparametric median and chi‐square tests with Monte Carlo method, which allows the estimation the exact significance when the necessary assumptions for the asymptotic method are not fulfilled. These tests helped to study possible significant differences regarding the length of hospitalisation period and clinical outcome among the three groups of patients hospitalized in the IDIU from first opinion or second opinion (appointments at the VTH and referred patients). Data analysis was conducted using IBM‐SPSS Statistics version 25.0.

## RESULTS

### Source of admitted patients

A total of 880 patients were admitted to the IDIU. Of the 365 dogs hospitalised at the unit during the study period, 67% were hospitalized after a first opinion appointment, 26% after referral and 7% after a second opinion appointment (Table 1).

**TABLE 1:** Median length of hospitalisation in days with numbers and minimum and maximum values (Min‐Max) for dogs and cats admitted to the IDIU by type of appointment for the period 2013–2018

		1st opinion patients				2nd opinion patients				Referred patients			
Species	Total N	N	(%)	Median	(Min‐Max)	N	(%)	Median	(Min‐Max)	N	(%)	Median	(Min‐Max)
Dogs	365	244	(66.8%)	3.0	(1–16)	25	(6.8%)	2.0	(1–7)	96	(26.3%)	3.0	(1–20)
Cats	515	400	(77.8%)	2.0	(1–21)	35	(6.8%)	2.0	(1–9)	80	(15.5%)	2.5	(1–27)
Totals	880	644	(73.2%)			60	(6.8%)			176	(20.0%)		

Regarding feline patients, of the 515 cats admitted to the unit, 78% were hospitalised after a first opinion appointment, 16% after referral and 7% after a second opinion appointment (Table 1).

**FIGURE 1:**
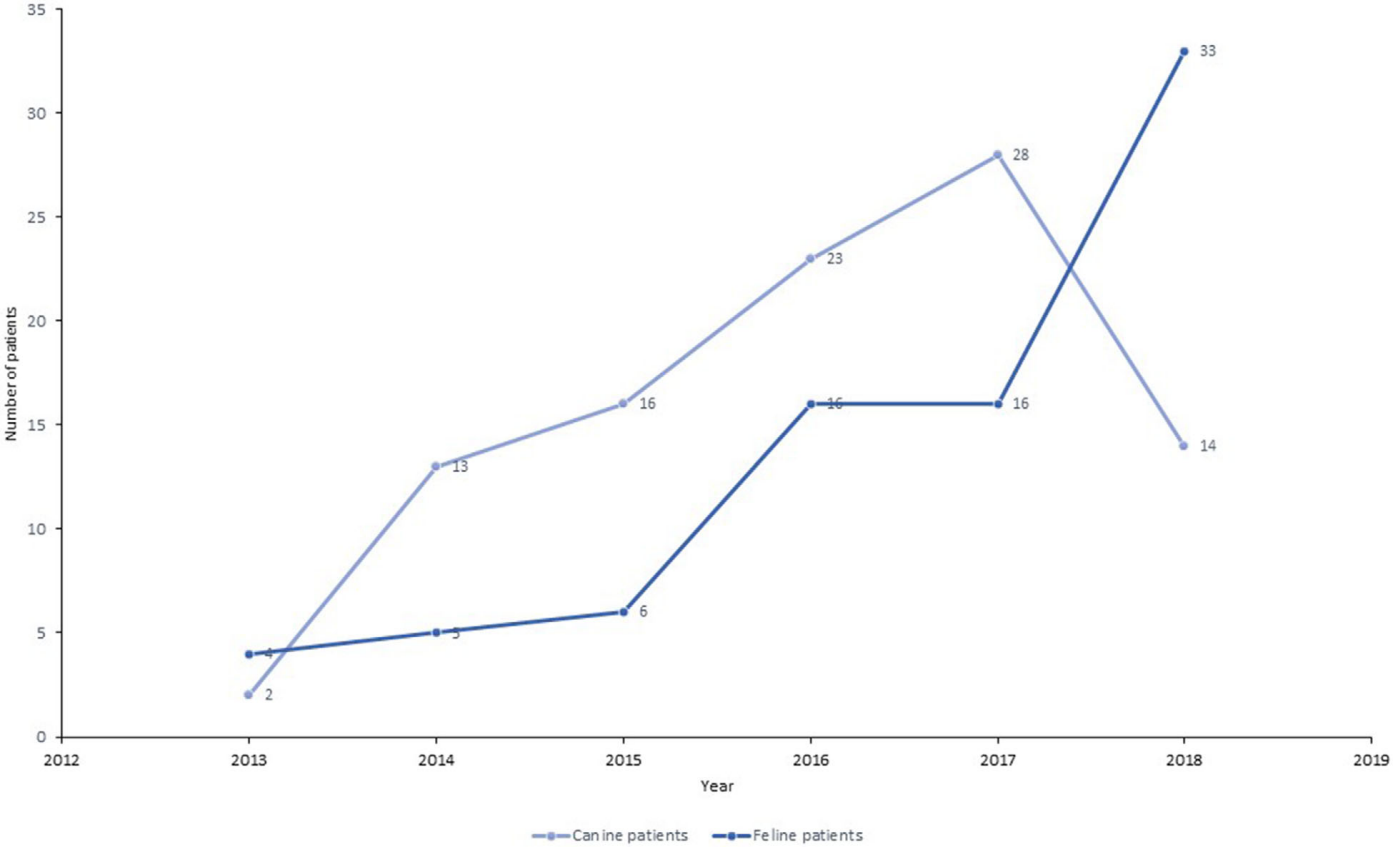
Annual numbers of referred patients to the IDIU in the period 2013–2018

There were an increasing number of patients referred to the unit each year since it opened in October 2013 to the end of December 2018 to give a total of 176 referred cases admitted during this 5‐year period (Figure 1). In canine patients, the peak of admissions was recorded in 2017 (N = 28), followed by a decrease to half that number (N = 14) in 2018. Regarding feline patients, there was a progressive increase in the number of cases per year between 2013 and 2016, from 4 to 16 cats. Then the number of referred feline patients remained the same in 2017 (N = 16) and achieved a peak in 2018 (N = 33).

### Animal species, sex, neuter status and age at admission of referred cases

Of the 176 patients referred to the unit, 55% (N = 96) were dogs, and 45% (N = 80) were cats. Half of the dogs were males, and 54% of the cats were males; most of the dogs were not neutered (N = 81, 84%), while more than half of the cats (N = 43, 54%) were neutered. The median age of dogs at admission was 2.0 years with the youngest puppy being 2.4 months and the oldest dog 15.0 years. Half of the dogs were juvenile (<1 year old) (N = 30, 32%) or young adults (≥1 to <3 years old) (N = 18, 19%). The median age of cats at admission was 6.0 years with a range from 1.2 months to 20.0 years, with about 30% of cats over 10 years old (N = 23).

### Vaccination status of referred cases

**FIGURE 2:**
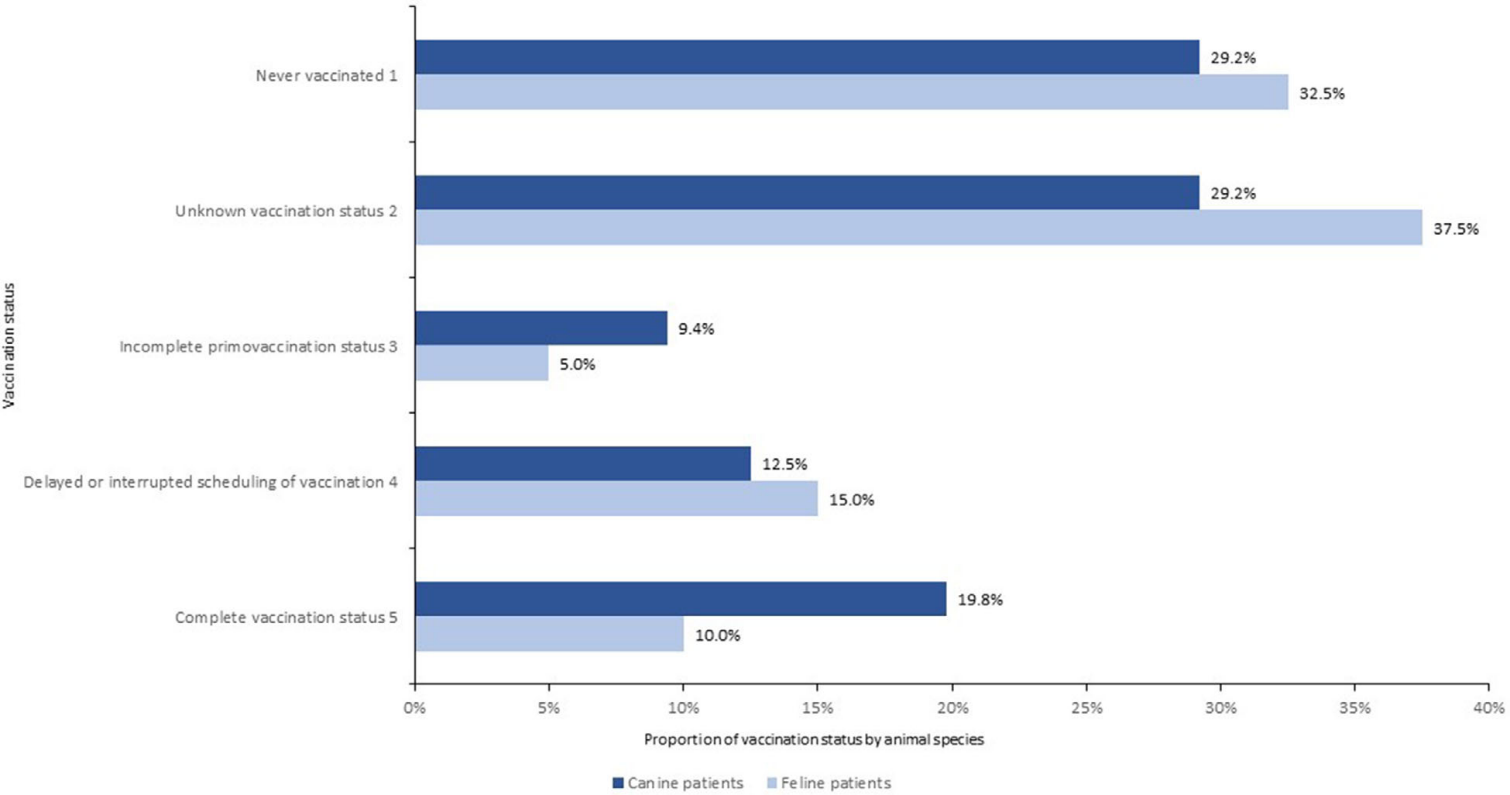
Vaccination status of canine and feline patients referred to the IDIU in the period 2013–2018. Never vaccinated^1^. Unknown vaccination status^2^. Incomplete primovaccination status^3^. Delayed or interrupted scheduling of vaccination^4^. Complete vaccination status^5^

Twenty‐nine per cent (N = 28) of the referred dogs had never received a vaccine, and the same proportion had an unknown vaccination status, while 9% (N = 9) had an incomplete primovaccination status and 13% (N = 12) a delayed or interrupted scheduling of vaccination, leaving only 20% (N = 19) having a complete vaccination schedule (Figure 2). Thirty‐three per cent (N = 26) of the referred cats never received a vaccine, 38% (N = 30) had an unknown vaccination status, 5% (N = 4) an incomplete primovaccination status and 15% (N = 12) a delayed or interrupted scheduling of vaccination, leaving only 10% (N = 8) having a complete vaccination schedule (Figure 2).

### Clinical presentation of referred cases

**FIGURE 3:**
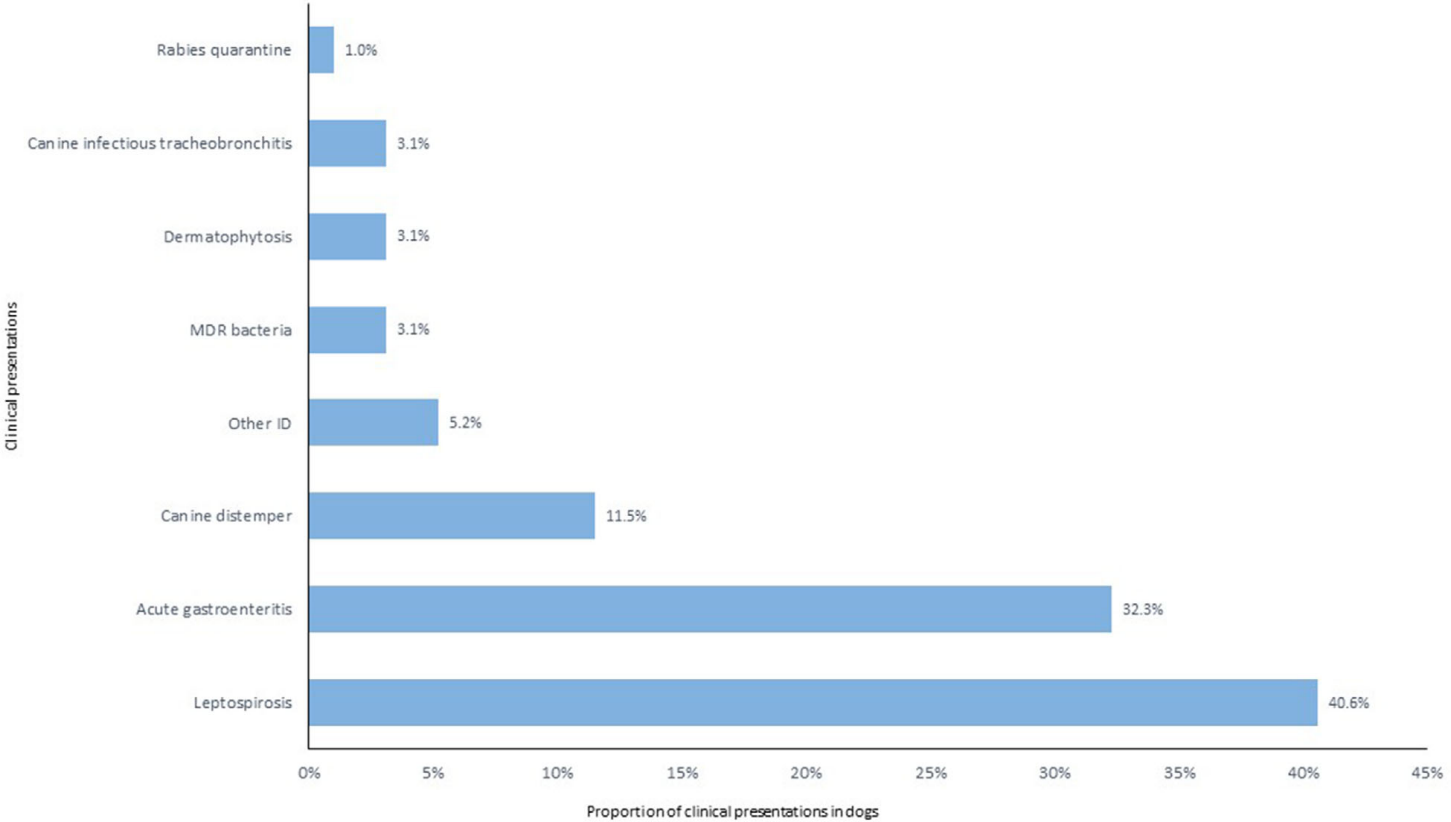
Clinical presentations of canine patients referred to the IDIU in the period 2013–2018 Abbreviations: Other ID, other infectious diseases; MDR bacteria, multidrug resistant bacteria.

**FIGURE 4:**
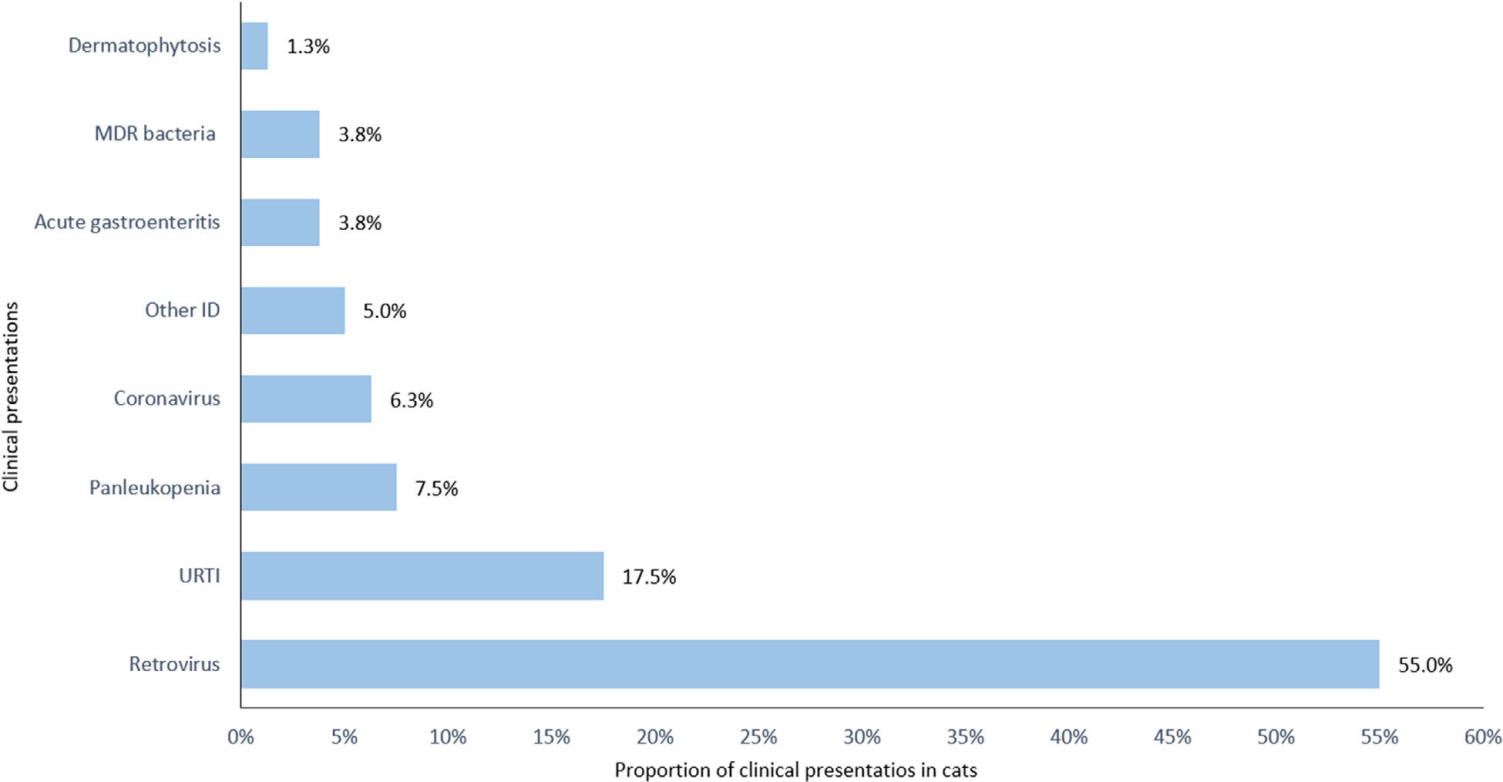
Clinical presentations of feline patients referred to the IDIU in the period 2013–2018 Abbreviations: Other ID, other infectious disease; URTI, Feline Upper Respiratory Tract Infection; MDR bacteria, multidrug resistant bacteria.

The three most frequent clinical presentations in referred dogs were compatible with canine leptospirosis (N = 39, 41%), followed by acute gastroenteritis (N = 31, 32%) and canine distemper (N = 11, 12%) as shown in Figure 3. In cats, the four main clinical presentations were compatible with retrovirus infections (N = 44, 55%), followed by feline upper respiratory tract infection (URTI, N = 14, 18%), feline panleukopenia (N = 6, 8%) and feline coronavirus (N = 5, 6%) as displayed in Figure 4.

### Definitive IDs diagnosis

**FIGURE 5:**
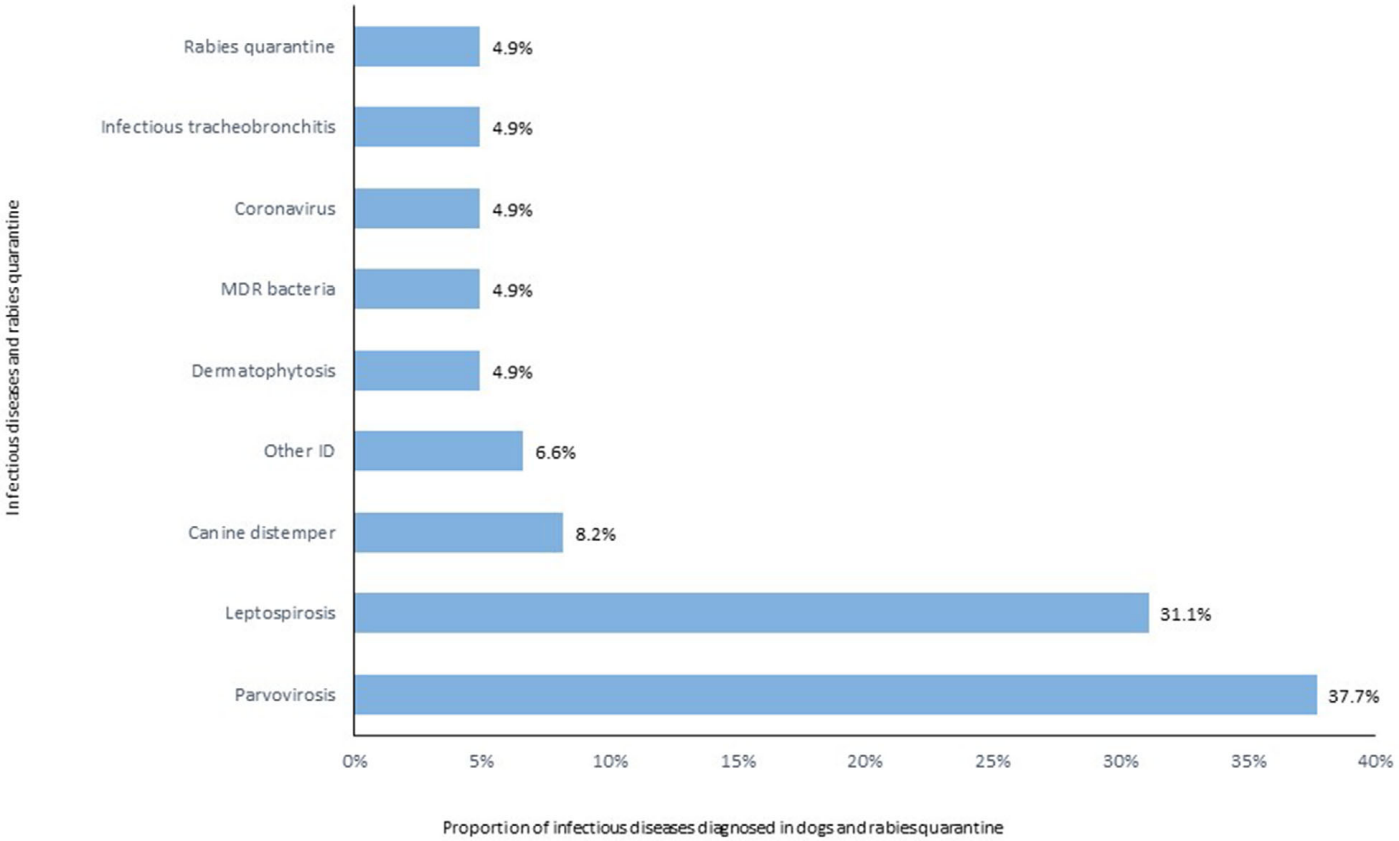
Infectious diseases diagnosed in canine patients referred to the IDIU in the period 2013–2018 including those admitted for rabies quarantine Abbreviations: Other ID, other infectious disease; MDR bacteria, multidrug resistant bacteria.

**FIGURE 6:**
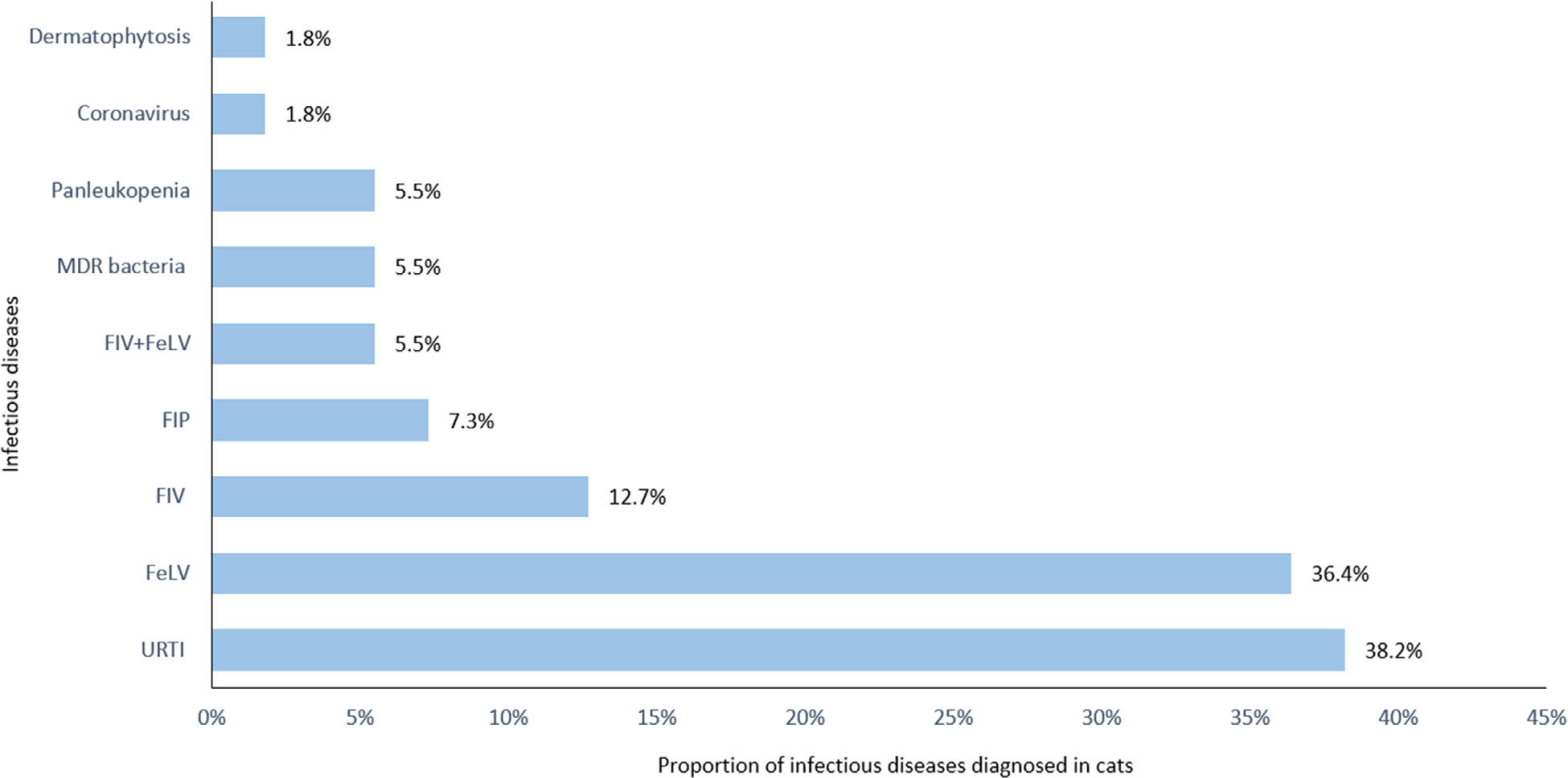
Infectious diseases diagnosed in feline patients referred to the IDIU in the period 2013–2018 Abbreviations: FeLV, feline leukaemia virus; FIP, feline infectious peritonitis; FIV, feline immunodeficiency virus; FIV+FeLV, cats infected with feline immunodeficiency virus and feline leukaemia virus; MDR, multidrug resistant bacteria; URTI, feline upper respiratory tract infection.

A positive ID definitive laboratory diagnosis was obtained in 64% of the canine patients (N = 61). Of the remaining 35 dogs, 16% (N = 15) were negative, and 21% (N = 20) remained suspected of having an ID. Three per cent (N = 3) dogs were positive for two or more ID. This situation accounted for 101 diagnoses. As shown in Figure 5, the most frequent ID diagnosed was parvovirosis (N = 23, 38%) followed by leptospirosis (N = 19, 31%). Among the feline referred patients, 69% (N = 55) had a definitive ID laboratory diagnosis with an additional 10% (N = 8) having two different ID diagnoses, and this increased the total number of ID diagnoses to 71. Twenty‐eight per cent (N = 22) remained suspected of having an ID, and only 4% of cats had negative test results (N = 3%). The most frequent feline ID was URTI (N = 21, 38%) followed by feline leukaemia virus (FeLV, N = 20, 36%) and FIV (N = 7, 12.7%, Figure 6).

### Concomitant non‐IDs

On the day of admission, 25% (N = 24) of referred dogs had concomitant non‐IDs. The most frequent concomitant diseases were haemoparasitosis and other parasitic diseases, orthopaedic, neurological and dermatological diseases. This scenario was worse for the feline patients as 68% (N = 54) of the cats had concomitant non‐IDs. Leading concomitant diseases in cats were urinary tract diseases, tumours, anaemias, orthopaedic, neurological and dermatological diseases. In this study the presence of concomitant non‐IDs did neither influence the length of the hospitalisation period nor the clinical outcome of referred patients (*p *= 0.13 for dogs and *p *= 0.7 for cats).

### Length of the hospitalisation period

The median hospitalisation time for the referred and first opinion dogs was the same at 3.0 days and was longer than that for the second opinion dogs at 2.0 days (Table 1), although these differences were not significant (χ^2^ [2, N = 365] = 5.66, *p* = 0.06). The median hospitalisation time was the same at 2.0 days for the first and second opinion patients and was shorter than that for the referred cats at 2.5 days, although these differences were not significant (χ^2^ [2, N = 514] = 1.31, *p *= 0.5).

**FIGURE 7:**
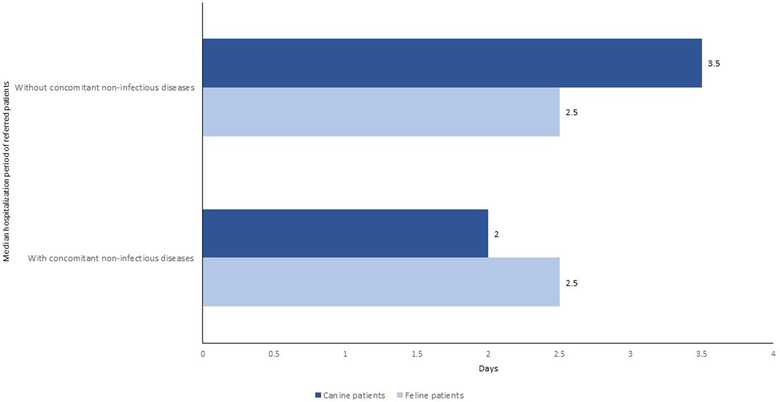
Median hospitalisation time in days of referred patients admitted to the IDIU with and without concomitant non‐infectious diseases in the period 2013–2018

The median hospitalisation time of 2.0 days for referred canine patients with concomitant non‐IDs was shorter than that of 3.5 days for dogs without concomitant non‐IDs (Figure 7), but the difference was not significant (χ^2^ [1, N = 96] = 3.159, *p = *0.12). The median hospitalisation time of 2.5 days for referred cats did not vary between patients with or without concomitant non‐IDs (Figure 7).

### Clinical outcome

In both species, the first opinion patients showed the highest proportion of patients discharged (79% of dogs and 77% of cats), followed by the referred patients (71% of dogs and 66% of cats) and finally by the second opinion patients (60% of dogs and cats (Table 2).

**TABLE 2:** Outcomes for dogs and cats admitted to the IDIU for the period 2013–2018, expressed as number (N) and per cent (%) of those admitted to the unit by source of patients

	First opinion		Second opinion		Referral		Total N	
Outcome in Dogs	N	(%)	N	(%)	N	(%)		(%)
Discharge	193	(79%)	15	(60%)	68	(71%)	275	(75%)
Euthanasia	27	(11%)	4	(16%)	13	(14%)	44	(12%)
Death	24	(10%)	6	(24%)	15	(16%)	46	(13%)
Total dogs	244	(100%)	25	(100%)	96	(100%)	365	(100%)

	First opinion		Second opinion		Referral		Total N	
Outcome in cats	N	(%)	N	(%)	N	(%)		(%)
Discharge	306	(77%)	21	(60%)	53	(66%)	380	(74%)
Euthanasia	61	(15%)	8	(23%)	18	(23%)	87	(17%)
Death	33	(8%)	6	(17%)	9	(11%)	48	(9%)
Total cats	400	(100%)	35	(100%)	80	(100%)	515	(100%)

The highest frequency of euthanasia was recorded for the second opinion patients (16% of dogs and 23% in cats) followed by the referred patients (14% of dogs and 23% of cats), and the lowest was in the first opinion patients (11% of dogs and 15% of cats). The highest mortality rate occurred in patients hospitalised following second opinion appointments for both species (24% of dogs and 17% of cats) followed by referred patients (16% of dogs and 11% of cats), and the lowest mortality rate was in the first opinion patients (10% of dogs and 8% of cats). The relationship between the types of patients and clinical outcome (discharge, death and euthanasia) was not significant for dogs or cats (χ^2^ [4, N = 365] = 7.02, *p* = 0.13; χ^2^ [4, N = 515] = 7.89, *p* = 0.10, respectively). However, the relationship between the types of patients and clinical outcome was significant for dogs and cats if the type of patients were contrasted between discharge versus died (including both death and euthanasia) (χ^2^ [2, N = 365] = 6.10, *p* = 0.05; χ^2^ (2, N = 515) = 7.31, *p* = 0.03, respectively). These results showed highest clinical discharge rates on the first opinion patients’ group (79%) followed by referred group for dogs (71%). In cats this difference was even higher comparing the first opinion patients’ (77%) with the referred group (66%). The second opinion groups had the lowest differences between discharge and death in both dogs and cat's patient groups (60% for both species).

## DISCUSSION

The total number of dogs referred to the IDIU increased steadily since its opening in 2013. A peak of referrals occurred in 2017 associated with dogs with clinical signs compatible with distemper. This was due to an outbreak of canine distemper clustered in the Lisbon Metropolitan Area in 2016–2017.^19^, ^20^ After that, the number of referred dogs decreased in 2018, approaching the frequency recorded before the distemper outbreak. The total number of cats referred to the unit stopped increasing in 2017, but then grew again to reach a peak in 2018. Referral of cats with clinical presentations compatible with feline retrovirus infection (55.0%) drove this growth.

The fact that almost 30% of the dogs admitted to the unit were <1 year of age is relevant to interpretation of the results as parvovirosis was the most frequently diagnosed ID in dogs. This finding is consistent with scientific knowledge of canine parvovirosis age distribution as it occurs manly in puppies aged between 6 weeks and 6 months^21^, ^22^, ^23^ and at the IDIU. Moreover the disease is widespread in Portugal, and the three known CPV‐2 variants circulate in the dog population.^21^

The age distribution of referred feline patients was also wide, and the right shift was mainly due to the hospitalisation of about 30% of geriatric cats (≥10 years old).

URTI and FeLV were the most frequent causes of hospitalisation of cats. In both diseases, kittens are much more susceptible to infection than adults are; however, these viruses may remain latent in host's tissues, and, during immunosuppression periods, virus may reactivate and induce clinical episodes of URI or FeLV at any stage of the cat's life span.^20^, ^24^, ^25^, ^26^, ^27^, ^28^, ^29^, ^30^ Two factors may explain the high frequency of URTI: the very low proportion of referred cats to the unit with a complete vaccination status (10%) and limitations of the current vaccines in preventing infections caused by FHV and FCV. These vaccines do not completely prevent vaccinated cats from becoming infected and from shedding these viruses after infection. In addition, there is no vaccine available in Europe that protects against all FCV field strains.^31^ The high frequency of FeLV is consistent with the results of a recent pan‐European study on the prevalence of FeLV in Southern Europe.^26^

In this study the presence of concomitant non‐IDs did not influence the length of hospitalisation or the clinical outcome of referred patients for dogs or cats.

The analysis of the length of hospitalisation stay in the three groups of patients—first opinion appointment, second opinion appointment and referred patients—revealed that referred dogs remained hospitalized for a similar period as the first opinion group (3 days), which was 1 day longer than the observed for the second opinion group (2 days), these differences were not statistically significant. Although not statistically significant the length of hospitalisation in referred cats suggest a tendency for half a day more (2.5 days) than the first and second opinion groups (2 days). Prolonged lengths of hospitalisation stay may be justified by the time taken to perform complex diagnostic exams and/or by performing for instance blood transfusions requested by the referring veterinarian.^10^, ^11^

The highest clinical discharge was observed on the first opinion patients’ group (79% in dogs and 77% in cats), suggesting that patients visiting the VTH for first‐option appointments received proper medical care in the right moment, allowing for their full recovery. The highest frequency of death occurred in patients hospitalised at IDIU by second opinion appointment (24% in dogs and 17% in cats), followed by the referred group (16% in dogs and 11% in cats). Comparing the types of patients and clinical outcome we found a significant difference between discharge versus died (including both death and euthanasia). These results showed highest clinical discharge rates on the first opinion patients’ group followed by referred group and lastly by the second opinion groups. This outcome was probably due to a combination of factors such as failure in receiving early medical care and poor prognosis on the day of admission, some being critically ill. Canine parvovirosis is a good illustration of this as this ID in the second opinion group caused half of the deaths. This ID requires a prompt and aggressive therapy to increase the survival rate of infected dogs^22^, ^23^, ^40^ that these patients lacked. In both species, euthanasia was more frequent in patients hospitalised by second opinion appointment (16% in dogs and 23% in cats), although these proportions are close to referred patients, especially in cats (14% in dogs and 23% in cats). The main causes of euthanasia were anuria in canine leptospirosis and sepsis in canine parvovirosis. Tumours and non‐reversible anaemia in FeLV infected cats were the major causes for euthanasia in feline patients.

## CONCLUSIONS

Veterinarian referring was mainly motivated due to clinical suspicion of parvovirosis and leptospirosis in dogs and URTI and FeLV in cats. Other major reasons for referring were a need for specialised medical assistance, definitive ID diagnosis or to mitigate the risk of ID spread in the clinics or hospital, especially when facing zoonosis.

We identified and quantified the most frequent ID in referred dogs and cats as well as their clinical outcomes. Serious non‐compliance in dog and cat vaccination schedules were discussed. To improve the vaccination coverage rate and to follow international expert opinion guidelines for the vaccination of dogs and cats should be a priority of all companion animal veterinarians working in Portugal.

Regarding the three groups of patients studied, the analysis of the length of hospitalisation did not reveal statistically significant differences, and further studies with a larger sample population may extend and better clarify these results.

In both animal species there were statistically significant higher clinical discharge rates on the first opinion patients’ group in comparison to referred patients and the second opinion group. These results suggest that patients visiting the VTH for first‐option appointments received proper medical care at the right moment, allowing for their full recovery.

This information will be very useful to make communication more assertive with referring veterinarians.

## ETHICS APPROVAL AND CONSENT TO PARTICIPATE

All animals that participated in this study were client‐owned and joined the study after owner's written consent and Ethical Committee approval.

## AVAILABILITY OF DATA AND MATERIAL

The datasets used and/or analyzed during the current study are available from the corresponding author on reasonable request.

## CONFLICT OF INTEREST

The authors declare that there is no conflict of interest that could be perceived as prejudicing the impartiality of the research reported.

## AUTHOR CONTRIBUTIONS

Catarina Paulo and Inês Machado analysed the data. Helena Carvalho performed the statistical analysis and helped drafting and revising the manuscript. Joana Gomes, Ana Deodato Mota, Luís Tavares and Virgílio Almeida helped to analyse the data. Solange Gil conceived the study and participated in its coordination, helped to draft the manuscript and supervised throughout. All authors read and approved the final manuscript.
